# High muscle‐to‐fat ratio is associated with lower risk of chronic kidney disease development

**DOI:** 10.1002/jcsm.12549

**Published:** 2020-02-05

**Authors:** Jong Hyun Jhee, Young Su Joo, Seong Hyeok Han, Tae‐Hyun Yoo, Shin‐Wook Kang, Jung Tak Park

**Affiliations:** ^1^ Division of Nephrology, Department of Internal Medicine Gangnam Severance Hospital, Yonsei University College of Medicine Seoul Korea; ^2^ Division of Nephrology, Department of Internal Medicine Myongji Hospital Goyang Gyeonggi‐do Korea; ^3^ Department of Internal Medicine, Institute of Kidney Disease Research Severance Hospital, Yonsei University College of Medicine Seoul Korea; ^4^ Department of Internal Medicine, College of Medicine, Severance Biomedical Science Institute, Brain Korea 21 PLUS Yonsei University Seoul Korea

**Keywords:** Muscle‐to‐fat ratio, Body mass index, Muscle mass, Fat mass, Obesity, Chronic kidney disease

## Abstract

**Background:**

Obesity, a known risk factor for chronic kidney disease (CKD), is generally assessed using body mass index (BMI). However, BMI may not effectively reflect body composition, and the impact of muscle‐to‐fat (MF) mass balance on kidney function has not been elucidated. This study evaluated the association between body muscle and fat mass balance, represented as the MF ratio, and incident CKD development.

**Methods:**

Data were retrieved from a prospective community‐based cohort study (Korean Genome and Epidemiology Study). Muscle and fat mass were measured using multifrequency bioelectrical impedance analysis. The study endpoint was incident CKD (estimated glomerular filtration rate <60 mL/min/1.73 m^2^ in at least two or more consecutive measurements during the follow‐up period).

**Results:**

Totally, 7682 participants were evaluated. Their mean age was 51.7 ± 8.7 years, and 48% of the subjects were men. During a median follow‐up of 140.0 (70.0–143.0) months, 633 (8.2%) subjects developed incident CKD. When the association between body composition and incident CKD was investigated, multivariable Cox proportional hazard analysis revealed that increase in MF ratio was related with a decreased risk of CKD development [per 1 increase in MF ratio: hazard ratio (HR), 0.86; 95% confidence interval (CI), 0.77–0.96; *P* = 0.008]. This association was also maintained when MF ratio was dichotomized according to sex‐specific median values (high vs. low: HR, 0.83; 95% CI, 0.70–0.98; *P* = 0.031). Analyses preformed in a propensity score matched group also revealed a similar decreased risk of incident CKD in high MF ratio participants (high vs. low: HR, 0.84; 95% CI, 0.71–0.98; *P* = 0.037). This relationship between MF ratio and incident CKD risk was consistently significant across subgroups stratified by age, sex, hypertension, estimated glomerular filtration rate categories, and proteinuria. Among different BMI groups (normal, overweight, and obese), the relationship between high MF ratio and lower incident CKD risk was significant only in overweight and obese subjects.

**Conclusions:**

Lower fat mass relative to muscle mass may lower the risk of CKD development in individuals with normal renal function. This relationship seems more prominent in overweight and obese subjects than in normal weight subjects.

## Introduction

Obesity is considered a major public health problem owing to its rapidly increasing prevalence and close association with poor outcomes.^1^ A commonly used measure for assessing adiposity is body mass index (BMI).^2^ Studies have shown BMI to be a significant risk factor for the development of various acute and chronic conditions such as hypertension, type 2 diabetes, cardiovascular diseases (CVDs), and certain types of cancer.^3^ Several recent large epidemiologic investigations have also reported that the risk of chronic kidney disease (CKD) development clearly increases with the increase in BMI.^4^ However, others have failed to observe such associations.^5^, ^6^

One major shortcoming of using BMI to determine obesity is that BMI does not account for body composition.^7^, ^8^ BMI, calculated based only on height and weight, is an inaccurate measure of body fat content and does not take into account the fluid status, muscle mass, or bone density.^9^, ^10^ Individuals with the same BMI may largely vary in body composition. This inaccuracy of BMI in representing adiposity may be one of the reasons for the controversial results among studies evaluating the association between BMI and CKD development.

Recent studies have suggested that body fat mass and muscle mass may differently affect health outcomes. An increase in muscle mass has been linked to less insulin resistance and protection against the development of type 2 diabetes.^11^, ^12^, ^13^ In addition, a lower amount of lean body mass has been reported to be related to an increased risk of cardiovascular events.^14^, ^15^ On the other hand, increased fat mass has been shown to increase the risk of metabolic derangements and poor cardiovascular outcome.^16^, ^17^ Moreover, the substantial loss of muscle mass relative to fat mass, termed as ‘sarcopenia’, has been found to have a negative effect on various cardiometabolic parameters and on mortality.^18^, ^19^, ^20^ This relationship has been observed not only in the elderly population and in patients with cancer, in whom muscle loss is prevalent, but also in the general population.^10^, ^21^ Nonetheless, the impact of muscle mass to fat mass balance on kidney function has not been clearly elucidated.

Therefore, the present study aimed to evaluate the association between the balance of body muscle and fat mass composition, represented as the muscle‐to‐fat (MF) ratio, and incident CKD development in a prospective community‐based cohort of subjects with normal renal function.

## Materials and methods

### Study subjects

This study used data from the Korean Genome and Epidemiology Study (KoGES), a prospective community‐based cohort study. The detailed profile and methods of how the KoGES cohort was assembled were previously described elsewhere.^22^ Briefly, the study cohort consisted of 10 030 subjects aged 40–69 years who were residents of Ansan or Ansung, which are cities near the capital city of Seoul, South Korea. The subjects underwent medical health evaluations and various surveys at baseline. Serial health examinations and surveys were performed biennially from 2001 to 2014. In this study, subjects with available body composition data were initially screened for inclusion. Subsequent exclusions were performed for those with an estimated glomerular filtration rate (eGFR) of <60 mL/min/1.73 m^2^ or underlying kidney disease at baseline, those with missing data, and those with missing follow‐up creatinine data. A total of 7682 subjects were included in the final analysis (Supporting Information, *Figure S1*). All subjects voluntarily participated in the study and provided informed consent. The study protocol was approved by the Ethics Committee of KoGES at the Korean National Institute of Health. This study was performed in accordance with the Declaration of Helsinki and approved by the institutional review board of Yonsei University Health System Clinical Trial Center (4‐2016‐0900).

### Anthropometric and laboratory data

All subjects underwent a comprehensive medical health examination and completed questionnaires about health and lifestyle factors at the time of study entry. Demographic and socio‐economic data, including age, sex, education and income level, smoking status, alcohol intake, and medical histories, were obtained. Anthropometric parameters such as height and weight were measured by trained researchers according to a specific protocol in which the subjects took off their shoes, wore light clothing, and stood on a scale on an even surface while measurements were taken. Education level was classified into three groups: low, lower than middle school; middle, middle school; and high, higher than middle school. Income level was divided into tertile groups based on the average per‐person monthly income: low, <$850 per month; middle, $850–1700 per month; and high, ≥$1700 per month. Physical activity status was categorized into active (at least 30 min/day of moderate‐intensity activity) or inactive. Subjects who had a blood pressure of ≥140/90 mmHg or were taking antihypertensive agents were considered to have hypertension. Those who had a blood glucose level of ≥126 mg/dL in an 8 h fasting status, had a post‐load glucose level of ≥200 mg/dL after a 75 g oral glucose tolerance test, had a haemoglobin A1c (HbA1c) value of ≥6.5%, or were taking oral medication and/or receiving insulin treatment for hyperglycaemia were considered to have diabetes. Subjects who had a medical history of dyslipidaemia or were taking medications for lipid control were considered to have dyslipidaemia. CVDs were defined as the composite of myocardial infarction, congestive heart failure, and coronary artery disease.

Blood and urine samples were obtained after an 8 h fast and transported to a central laboratory (Seoul Clinical Laboratories, Seoul, Republic of Korea) within 24 h of sampling. The serum concentrations of blood urea nitrogen, creatinine, albumin, glucose, total cholesterol, triglyceride, high‐density lipoprotein cholesterol (HDL‐C), and C‐reactive protein (CRP) were measured using ADVIA 1650 (Siemens, Tarrytown, NY, USA). Serum creatinine level was measured using the Jaffé method throughout the study period. The creatinine levels were reduced by a calibration factor of 5% for standardization to isotope dilution mass spectrometry reference method values.^23^ Low‐density lipoprotein cholesterol (LDL‐C) level was calculated using the following formula: [total cholesterol (mg/dL) − HDL‐C (mg/dL) − triglyceride (mg/dL) /5]. The HbA1c level was determined using high‐performance liquid chromatography (Variant II; BioRad Laboratories, Hercules, CA, USA). Haemoglobin levels were measured using an autoanalyser (Sysmex, Kobe, Japan). Homeostatic model assessment of insulin resistance (HOMA‐IR) was calculated by following formula: [fasting insulin (uU/mL) × fasting plasma glucose (mg/dL) /405]. Urine samples were collected in the morning after the first voiding and subjected to a dipstick test using URISCAN Pro II (YD Diagnostics Corp., Seoul, Korea). Proteinuria was quantified as absent, trace, 1+, 2+, or 3+ based on a colour scale. The presence of proteinuria was defined as a dipstick urine test result of more than or equal to the trace level.

### Assessment of body composition

Body composition was assessed using multifrequency bioelectrical impedance analysis (BIA; InBody 3.0, Biospace, Seoul, Korea). Compared with conventional BIA‐based analysis methods that rely on formulae to calculate the estimated mass of each body component, multifrequency BIA assumes that the human body consists of five interconnecting cylinders and performs impedance measurements directly on these compartments. Using a tetrapolar 8‐point tactile electrode system, impedances were measured at four specific frequencies (5, 50, 250, and 500 kHz) in five segments (right arm, left arm, trunk, right leg, and left leg). Muscle mass and fat mass were expressed as the muscle mass index (MMI, muscle mass/height^2^) and the fat mass index (FMI, fat mass/height^2^), respectively. The MF ratio was defined as MMI/FMI. BMI was calculated as weight divided by height squared (kg/m^2^). Subjects were allocated into two groups based on the sex‐specific median values of the MF ratio. Sex‐specific median value of the MF ratio was defined as the median of the MF ratio in men and women, separately. The World Health Organization obesity classification for Asian populations was used to determine overweight and obesity.

### Study outcome

The primary endpoint was incident CKD development. CKD was defined as an eGFR of <60 mL/min/1.73 m^2^ in at least two or more consecutive measurements during the follow‐up period. The eGFR was calculated using the CKD Epidemiology Collaboration equation.^24^

### Statistical analysis

All statistical analyses were performed using IBM SPSS software for Windows version 23.0 (IBM Corporation, Armonk, NY, USA), SAS software version 9.2 (SAS Institute Inc., Cary, NC, USA), and R software 3.3.1 (http://www.R‐project.org). Continuous variables were expressed as means ± standard deviations, and categorical variables were expressed as absolute numbers with percentages. All data were tested for normality before statistical analysis. The Kolmogorov–Smirnov test was performed to determine the normality of the distribution of the parameters. Intergroup comparisons were performed using analysis of variance or Student's *t*‐test for normally distributed continuous variables, whereas categorical variables were examined using the *χ* ^2^ test or Fisher's exact test. Data that did not show normal distribution were presented as medians with interquartile ranges and compared using the Mann–Whitney *U* test or Kruskal–Wallis test. Cumulative renal survival rates were estimated using Kaplan–Meier analyses and log‐rank tests. Survival time was defined as the time interval between baseline and the onset of outcome or the last follow‐up. Subjects who were lost during follow‐up were censored in the final analysis. Cox proportional hazard models were constructed to determine the independent predictive value of the MF ratio for incident CKD development. Variables that presented statistical significance in the univariable analysis were included in the multivariable models (Supporting Information, *Table* *S1*). Model 1 was not adjusted for any covariates. Model 2 included baseline age and sex. Model 3 included demographic factors and co‐morbidities. Model 4 was further adjusted for laboratory parameters. For the propensity score matching (PSM) analyses, the propensity score was determined using binary logistic regression with greedy nearest neighbour matching technique without replacement. A caliper of 0.2 times the standard deviation was used. Subjects with high MF ratio were matched to those with low MF ratio. In the matched cohort, the groups were compared with paired *t*‐test and McNemar test, as appropriate. To test the relationship between incident CKD risk and MF ratio as a continuous variable, restricted cubic spline analyses were conducted. Extreme outliers, defined as values less than first quartile (Q1) − 1.5 × interquartile range (Q3–Q1) or greater than third quartile (Q3) + 1.5 × interquartile range, were excluded when depicting the cubic spline analysis results. For sensitivity analysis, analyses were conducted with creatinine levels adjusted through a conversion equation different from the formula used in the main analyses.^25^, ^26^ *P*‐values of <0.05 were considered statistically significant.

## Results

### Baseline characteristics

The baseline characteristics of the study subjects according to the sex‐specific median values of the MF ratio are described in *Table* 1. The median MF ratio was 3.4 [2.8–4.2] and 2.0 [1.7–2.3] in male and female participants, respectively. The mean age was 51.4 ± 8.7 years, and 3686 (48%) subjects were men. The mean eGFR was 93.9 ± 14.2 mL/min/1.73 m^2^. The MMI, FMI, BMI, and waist‐to‐hip ratio were significantly lower in the high MF ratio group than in the low MF ratio group. Subjects in the high MF ratio group were younger and were more likely to be physically active than those in the low MF ratio group. However, the proportions of men, smokers, and alcohol drinkers were comparable between the groups. Subjects in the high MF ratio group were more likely to engage in physical activities than those in the low MF ratio group. The systolic blood pressure (SBP) was lower, and fewer subjects had co‐morbidities such as hypertension, diabetes, dyslipidaemia, and CVDs in the high MF ratio group than in the low MF ratio group. With respect to laboratory test results, eGFR was higher and the proportion of proteinuria‐positive subjects was lower in the high MF ratio group than in the low MF ratio group. The levels of haemoglobin, albumin, total cholesterol, LDL‐C, triglyceride, fasting plasma glucose, HbA1c, HOMA‐IR, and CRP were significantly lower in the high MF ratio group than in the low MF ratio group.

**Table 1:** Baseline characteristics according to sex‐specific median of the muscle‐to‐fat ratio

Characteristics	Sex‐specific MF ratio			
	Total (*n* = 7682)	Low (*n* = 3839)	High (*n* = 3843)	*P*
Body composition				
MF ratio	2.9 ± 1.2	2.2 ± 0.6	3.5 ± 1.3	<0.001
Muscle mass index (kg/m^2^)	16.8 ± 1.7	17.2 ± 1.7	16.4 ± 1.7	<0.001
Fat mass index (kg/m^2^)	6.8 ± 2.4	8.3 ± 2.1	5.2 ± 1.6	<0.001
BMI (kg/m^2^)	24.6 ± 3.1	26.5 ± 2.6	22.7 ± 2.2	<0.001
WHR	0.87 ± 0.08	0.90 ± 0.07	0.85 ± 0.07	<0.001
Demographic data				
Age (years)	51.4 ± 8.7	52.3 ± 8.7	50.4 ± 8.6	<0.001
Male, *n* (%)	3686 (48.0)	1846 (48.1)	1840 (47.9)	0.437
Smoking status, *n* (%)	3129 (41.2)	1533 (40.4)	1596 (42.0)	0.083
Alcohol status, *n* (%)	4143 (54.4)	2050 (53.8)	2093 (54.9)	0.173
Physical activity status, *n* (%)	3796 (50.7)	1821 (48.6)	1975 (52.8)	<0.001
Physical activity (Mets)	9095.1 ± 6037.2	8492.5 ± 5736.9	9697.1 ± 6266.1	<0.001
SBP (mmHg)	121.0 ± 18.4	124.3 ± 18.7	117.6 ± 17.5	<0.001
Education, *n* (%)				<0.001
Low	2282 (29.9)	1223 (32.1)	1059 (27.7)	
Intermediate	4214 (55.3)	1999 (52.5)	2215 (58.0)	
High	1130 (14.8)	584 (15.3)	546 (14.3)	
Income, *n* (%)				0.372
Low	2357 (31.2)	1196 (31.7)	1161 (30.6)	
Intermediate	2185 (28.9)	1064 (28.2)	1121 (29.6)	
High	3019 (39.9)	1512 (40.1)	1507 (39.8)	
Co‐morbidities, *n* (%)				
Hypertension	1071 (13.9)	740 (19.3)	331 (8.6)	<0.001
Diabetes	493 (6.4)	294 (7.7)	199 (5.2)	<0.001
Dyslipidaemia	203 (2.6)	122 (3.2)	81 (2.1)	0.002
CVDs	105 (1.4)	70 (1.8)	35 (0.9)	<0.001
Laboratory data				
eGFR (mL/min/1.73 m^2^)	93.9 ± 14.2	92.9 ± 14.1	94.9 ± 14.3	<0.001
Proteinuria (%)	581 (7.6)	321 (8.4)	260 (6.8)	0.005
Haemoglobin (g/dL)	13.6 ± 1.6	13.8 ± 1.5	13.4 ± 1.6	<0.001
Albumin (g/dL)	4.52 ± 0.28	4.53 ± 0.28	4.51 ± 0.29	0.003
Total cholesterol (mg/dL)	199.1 ± 36.7	206.3 ± 36.9	191.8 ± 35.1	<0.001
LDL‐C (mg/dL)	119.2 ± 34.5	124.1 ± 35.0	114.2 ± 33.2	<0.001
HDL‐C (mg/dL)	49.5 ± 11.8	47.5 ± 10.7	51.6 ± 12.4	<0.001
Triglyceride (mg/dL)	151.8 ± 108.3	173.5 ± 119.0	130.1 ± 91.3	<0.001
Fasting glucose (mg/dL)	92.6 ± 23.2	95.2 ± 24.7	90.0 ± 21.2	<0.001
HbA1c (%)	5.8 ± 0.9	5.9 ± 0.9	5.7 ± 0.8	<0.001
HOMA‐IR	1.7 ± 1.2	2.0 ± 1.4	1.5 ± 1.1	<0.001
CRP [IQR] (mg/dL)	0.14 [0.06–0.25]	0.17 [0.09–0.28]	0.12 [0.05–0.20]	<0.001

Data are presented as mean (standard deviation), median [interquartile range], or number (%). Sex‐specific median of the MF ratio was 3.4 [2.8–4.2] in men and 2.0 [1.7–2.3] in women. BMI, body mass index; CRP, C‐reactive protein; CVDs, cardiovascular diseases; eGFR, estimated glomerular filtration rate; HDL‐C, high‐density lipoprotein cholesterol; HbA1c, haemoglobin A1c; HOMA‐IR, homeostatic model assessment of insulin resistance; LDL‐C, low‐density lipoprotein cholesterol; MF ratio, muscle‐to‐fat ratio; SBP, systolic blood pressure; WHR, waist‐to‐hip ratio.

**Table 2:** Risk of chronic kidney disease development according to body composition indices

	Model 1		Model 2		Model 3		Model 4	
	HR (95% CI)	*P*	HR (95% CI)	*P*	HR (95% CI)	*P*	HR (95% CI)	*P*
BMI								
Per 1 kg/m^2^ increase	1.08 (1.05–1.11)	<0.001	1.08 (1.06–1.11)	<0.001	1.05 (1.02–1.08)	<0.001	1.04 (0.99–1.08)	0.059
High vs. low	1.37 (1.17–1.60)	<0.001	1.48 (1.26–1.73)	<0.001	1.22 (1.04–1.45)	0.017	1.10 (0.93–1.31)	0.245
MF ratio								
Per 1 increase	0.75 (0.69–0.82)	<0.001	0.74 (0.67–0.82)	<0.001	0.82 (0.74–0.91)	<0.001	0.86 (0.77–0.96)	0.008
High vs. low	0.68 (0.58–0.80)	<0.001	0.68 (0.58–0.79)	<0.001	0.80 (0.67–0.94)	0.007	0.83 (0.70–0.98)	0.031

Model 1: unadjusted model; Model 2: adjusted for age and sex; Model 3: adjusted for Model 2 + systolic blood pressure, smoking status, alcohol intake, education levels, income levels, history of hypertension or diabetes, and physical activity; and Model 4: adjusted for Model 3 + estimated glomerular filtration rate, proteinuria, total cholesterol, and C‐reactive protein. BMI, body mass index; CI, confidence interval; HR, hazard ratio; MF ratio, muscle‐to‐fat ratio.

When the baseline characteristics were compared between male and female participants, male subjects were younger. In addition, male participants had a higher MF‐ratio, MMI, and WHR. The FMI and BMI were lower in males compared to female participants. Male participants were more likely to smoke and drink alcohol and have a higher education and income than female participants. In addition, hypertension was more common while diabetes and dyslipidaemia were less common among men than women. Regarding laboratory test results, eGFR, LDL‐C, and HDL‐C levels were lower while haemoglobin, serum albumin, triglyceride, fasting glucose, HOMA‐IR, and CRP levels were higher among men than among women (Supporting Information, *Table S2*).

### Relationship between muscle‐to‐fat ratio and metabolic factors

The associations between body composition indices and metabolic variables are shown in Supporting Information, *Table S3*. BMI revealed a positive relationship with SBP and total cholesterol, LDL‐C, triglyceride, HOMA‐IR, and CRP levels, whereas age and eGFR were negatively associated with each other. The MF ratio showed a positive association with serum albumin level, whereas a negative association was found among age, SBP, eGFR, and total cholesterol, LDL‐C, triglyceride, HOMA‐IR, and CRP levels.

When the relationship between body composition components and BMI was evaluated, a strong negative correlation was found between the MF ratio and BMI (*β* = −0.560, Supporting Information, *Figure S2A*). However, both the MMI and FMI showed a positive association with BMI (*β* = 0.618 and *β* = 0.809, respectively; Supporting Information, *Figure S2B* and *S2C*).

### Association between muscle‐to‐fat ratio and chronic kidney disease development

During a median follow‐up of 140.0 (70.0–143.0) months, 633 (8.2%) subjects developed incident CKD. The follow‐up duration was similar between the high and low MF ratio groups [140.0 (69.9–143.0) vs. 141.0 (71.0–143.1) months, *P* = 0.140]. Cox proportional hazard analyses were performed to assess the association between the MF ratio and CKD development. When BMI was dichotomized according to the sex‐specific median values, CKD development risk was significantly increased in the high BMI group compared with the low BMI group in the unadjusted model [hazard ratio (HR), 1.37; 95% confidence interval (CI), 1.17–1.60]. However, when adjustments were made for confounding factors, the elevated CKD risk in the high BMI group was no longer significant (HR, 1.10; 95% CI, 0.93–1.31). On the other hand, CKD development risk was significantly decreased in the high MF ratio group than in the low MF ratio group, even after adjusting for confounding variables (HR, 0.83; 95% CI, 0.70–0.98). A similar association was found when MF ratio was considered as a continuous variable (*Table* 2). When restricted cubic spline analyses were further conducted, CKD development risk was noted to gradually decrease significantly with the increase of MF ratio (Supporting Information, *Figure S3*).

In order to further minimize the effect of confounding factors, the risk for incident CKD was further determined in 3280 participants matched by propensity score. As shown in Supporting Information, *Table S4*, the high and low MF ratio groups were well matched for baseline characteristics after PSM. Cox proportional hazard analyses preformed in the PSM group revealed that participants with a high MF ratio were associated with a significantly decreased risk of incident CKD compared with those with a low MF ratio (HR, 0.84; 95% CI, 0.71–0.98), a finding consistent with the result of the non‐matched cohort (Supporting Information, *Table S5*).

For sensitivity analysis, evaluations were also performed with creatinine levels that were adjusted using a conversion equation different from the formula used in the main analyses. The sensitivity analysis results were consistent with the main findings of the study (Supporting Information, *Table S6*).

### Relationship of muscle‐to‐fat ratio with chronic kidney disease incidence in different body mass index groups

**Figure 1:**
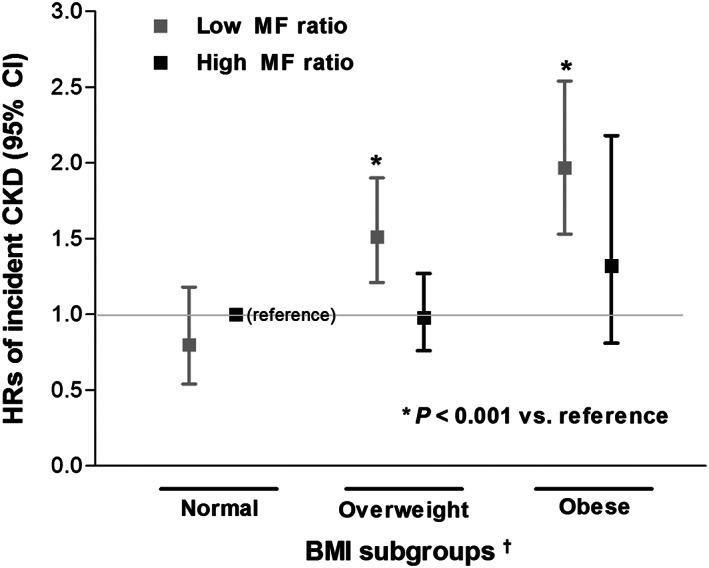
Comparison of risk for CKD development according to combination of BMI and sex‐specific median values of the MF ratio (low BMI with high MF ratio as reference group). BMI, body mass index; CI, confidence interval; CKD, chronic kidney disease; HRs, hazard ratios; MF ratio, muscle‐to‐fat ratio. ^†^World Health Organization obesity classification for Asian population was used: normal (BMI <23.0 kg/m^2^), overweight (BMI 23.0–27.4 kg/m^2^), and obese (BMI ≥27.5 kg/m^2^).

**Figure 2:**
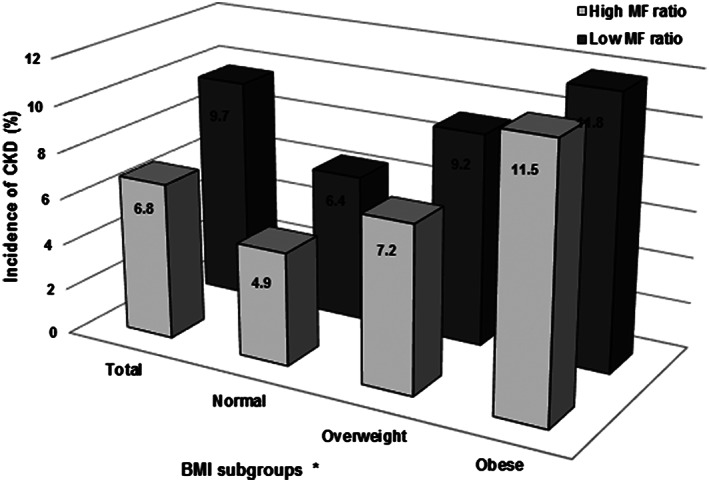
The prevalence of CKD according to sex‐specific median of the MF ratio in different BMI groups (*P* for trend <0.001). BMI, body mass index; CKD, chronic kidney disease; MF ratio, muscle‐to‐fat ratio. ^*^World Health Organization obesity classification for Asian population was used: normal (BMI <23.0 kg/m^2^), overweight (BMI 23.0–27.4 kg/m^2^), and obese (BMI ≥27.5 kg/m^2^).

Further evaluations were made to assess the impact of the MF ratio in overweight and obese subgroups classified based on the BMI criteria. When the subjects were classified into normal weight (BMI <23.0 kg/m^2^), overweight (BMI 23.0–27.4 kg/m^2^), and obese (BMI ≥27.5 kg/m^2^) groups, a significant gradual increase in the risk of incident CKD development was found in those with a low MF ratio. However, in those with a high MF ratio, the risk of CKD development in overweight and obese subjects was comparable with that in subjects with normal BMI (*Figure* 1). In addition, the time to CKD development was significantly longer in those with a high MF ratio. This advantage against incident CKD development was maintained in overweight and obese subjects, but not in those with a normal BMI (Supporting Information, *Figure S4*). When the incidences of CKD were stratified against the sex‐specific median values of the MF ratio and the BMI group, the CKD incidence rate was the highest in obese subjects with a low MF ratio and the lowest in those with normal BMI and a high MF ratio (*Figure* 2).

### Subgroup analysis

The relationship between the MF ratio and incident CKD was further evaluated in subgroups stratified by age (<50 vs. ≥50 years), sex (female vs. male), hypertension (yes vs. no), eGFR (60–79.9 vs. 80–99.9 vs. ≥100 mL/min/1.73 m^2^), and proteinuria (yes vs. no). No significant interactions were found in any of the subgroups, suggesting that the relation between the MF ratio and incident CKD risk was consistently significant across these subgroups (Supporting Information, *Figure S5*).

### Follow‐up loss

The overall follow‐up loss rate during the whole study duration was 29.5%. However, more than 62% of the subjects remained in the study for over 125 months (Supporting Information, *Figure S6*). The proportion of subjects lost to follow‐up was comparable between the high and low MF ratio groups [1170 (30.5%) vs. 1093 (28.4%), *P* = 0.051]. Changes in address or telephone number, being too busy to attend, and not answering telephone calls were the main reasons of follow‐up loss. Cumulative death rate between the groups was also comparable [367 (9.6%) vs. 336 (8.7%), *P* = 0.114].

## Discussion

In this study, the association of BMI and the MF ratio with CKD development was investigated in a general population cohort with preserved kidney function. Metabolic indices were positively correlated with BMI and negatively correlated to the MF ratio. BMI did not show a significant relationship with CKD development after full adjustment, whereas the increase in the MF ratio was clearly associated with a decreased risk of CKD development. In addition, the impact of the MF ratio on CKD development was more definite in overweight or obese subjects than in those with a normal BMI.

Previous studies evaluating the effect of adiposity on CKD development have shown conflicting results. Obesity defined by BMI was significantly associated with an increased risk of incident CKD in most of the studies. This association was observed not only in cases when increased BMI was accompanied by deranged metabolic features such as dyslipidaemia and diabetes but also in metabolically healthy individuals who do not have features of metabolic disease other than being overweight.^27^, ^28^, ^29^ However, several other studies failed to show such a relationship. In a prospective cohort study (the Framingham Offspring Study), increased BMI itself was not an independent risk factor for stage 3 CKD development.^6^ In addition, an observational study of Japanese subjects without kidney disease showed that obese individuals without metabolic complications were not exposed to a higher risk of incident CKD compared with non‐obese healthy subjects.^30^ These controversies could be based on the fact that BMI is a surrogate measure with considerable limitations in assessing adiposity.^7^, ^8^ As BMI is not a surrogate of fat mass alone but represents the composite of fat, muscle, bone, and fluid, assessing the effect of each component of BMI is not possible.^31^ This limitation of BMI should be further emphasized on the basis of recent reports showing that excessive adiposity is metabolically harmful whereas muscle mass plays a beneficial role. The findings of this study demonstrating that BMI correlates positively with the FMI but negatively with the MF ratio support the notion that the associations of BMI with body composition factors could be diverse.

The impact of the MF ratio on outcome has been previously evaluated in patients with high‐risk factors for sarcopenia, such as advanced CKD.^14^ By evaluating patients with stage 3–5 CKD not yet treated with dialysis, Lin *et al*. showed that lean tissue index rather than BMI alone provided better risk prediction of cardiovascular outcome in these patients. Moreover, they found that a high lean/fat tissue index was associated with the best outcomes. Another recent report showed that in patients undergoing maintenance haemodialysis, the mortality risk is increased in those with a high BMI and increased body fat but decreased in those with a high BMI and high muscle mass.^32^ Despite these previous reports, studies evaluating the effect of body composition on renal outcome in relatively healthy subjects with preserved renal function are lacking. The results of the present study suggest that the balance of fat mass and muscle mass could be a considerable factor when assessing the risk of kidney function decline in the general population.

Several possible mechanisms by which the MF ratio affects kidney function can be postulated. Decrease in muscle mass is frequently linked with chronic inflammation.^33^ Increased low‐grade systemic inflammation or pro‐inflammatory cytokines related to low muscle mass may consequently induce endothelial dysfunction, leading to a decrease in kidney function.^34^, ^35^ In addition, skeletal muscle is the primary site of insulin‐mediated glucose uptake. A previous report has shown that greater muscle mass is clearly associated with increased insulin sensitivity.^36^ As insulin resistance and related metabolic complications are well‐known factors that damage the kidneys, healthier metabolic features associated with increased muscle mass can contribute to preventing kidney function decline. The circulating levels of fasting glucose, HbA1c, and HOMA‐IR were significantly lower in subjects with a high MF ratio in this study, supporting this possibility. Furthermore, recent studies have suggested that the skeletal muscle is an endocrine organ. Myokines, which are peptides released from the skeletal muscle, have been observed to affect kidney function, a phenomenon proposed as muscle–kidney crosstalk.^37^ A well‐recognized myokine, irisin, has been demonstrated to improve kidney energy metabolism and prevent kidney damage in mice. On the other hand, increased fat mass is associated with chronic inflammation and induces negative metabolic effects such as impaired glucose tolerance and deranged lipid profile.^38^ These increased inflammation and negative metabolic effects may lead to structural changes in the kidney, such as mesangial expansion and renal fibrosis.^39^ Supporting this notion, the present study demonstrated that the levels of CRP, LDL‐C, and triglyceride were significantly higher in subjects with a low MF ratio. In addition, increased fat mass may confer adverse haemodynamic effects on the kidney, inducing higher filtration fraction and increased glomerular capillary pressure.^40^ Considering these beneficial and adverse properties of muscle and fat mass, the net additive effect of the balance between the two body components could have an impact on kidney function.

This study has several limitations. First, body composition was measured using BIA only. Dual‐energy X‐ray absorptiometry is also considered a reliable method for body composition assessment.^41^ Nevertheless, previous reports have confirmed that multifrequency BIA systems can provide accurate muscle mass and fat mass values that are comparable with those measured using dual‐energy X‐ray absorptiometry in various populations.^42^, ^43^ In addition, the Asian Working Group of Sarcopenia supports the use of BIA for body composition assessment in community‐based settings, owing to its simplicity and reliability.^44^ Second, a single measurement of body composition at baseline was used for the analysis. As the body composition changes over time, values obtained during a longer follow‐up period may provide a more accurate body composition status. Further investigations considering body composition indices as time‐dependent factors would be needed. Third, evaluations among underweight individuals were not possible due to the small size of this group (120, 1.6%). Further assessments would be needed to verify whether the findings of this study are maintained among those with low BMI. Fourth, although the creatinine levels had been standardized to isotope dilution mass spectrometry reference method values through two different calibration formulae, possibilities of bias still remain. Lastly, owing to the observational study design, a clear causal relationship between a high MF ratio and a lower risk of incident CKD could not be established. Nevertheless, a high MF ratio was independently associated with an increased risk of CKD development even after adjustments for extensive covariates, lowering the chance of bias.

In conclusion, a high MF ratio is associated with a decreased risk of incident CKD in subjects with normal kidney function. This association is more prominent in overweight‐to‐obese subjects. Therefore, in addition to body mass, the balance between body composition components would need to be considered when stratifying the risk of CKD development.

## Author contributions

J.J.H. and J.T.P. researched data. J.J.H. wrote the manuscript and researched data. J.T.P. reviewed and edited the manuscript. Y.S.J. contributed to the discussion and reviewed and edited the manuscript. S.D.H., S.H.H., T.H.Y., J.H.S., S.W.L., and S.W.K. researched data and contributed to the discussion. J.T.P. is the guarantor for this work and takes responsibility for the integrity of the data. All authors critically revised the manuscript for key intellectual content and approved the final version of the manuscript. The authors certify that they comply with the ethical guidelines for publishing in the *Journal of Cachexia, Sarcopenia and Muscle*.^45^

## Conflict of interest

None declared.

## Supporting information


**Table S1.** Risk of CKD development according to baseline characteristics
**Table S2.** Baseline characteristics (Male vs. Female)
**Table S3.** Univariable correlations between components of body composition and baseline characteristics
**Table S4.** Baseline characteristics according to sex‐specific median of the MF‐ratio after propensity score matching
**Table S5.** Risk of CKD development according to body composition indices after propensity score matching
**Table S6.**Sensitivity analysis: Risk of CKD development according to body composition indices using different creatinine conversion formula
**Figure S1.**Study subjects
**Figure S2.**Mutual relationships among components of body composition
**Figure S3.** Restricted cubic spline plot for incident CKD according to MF‐ratio
**Figure S4.** Cumulative Hazards for the incident CKD development according to sex‐specific median of the MF‐ratio in all study subjects (A), normal BMI group (B), overweight group (C), and obese group (D)
**Figure S5.** Subgroup analyses of risk for incident CKD according to high vs. low MF‐ratio groups
**Figure S6.**Frequency of cumulative study visit months
