# Supplementary material for: High muscle‐to‐fat ratio is associated with lower risk of chronic kidney disease development
Source: J Cachexia Sarcopenia Muscle. 2020 Feb 5;11(3):726–34. doi: 10.1002/jcsm.12549 (PMC7296269; doi:10.1002/jcsm.12549)
Supplement: Supplementary file 1 — Table S1. Risk of CKD development according to baseline characteristics Table S2. Baseline characteristics (Male vs. Female) Table S3. Univariable correlations between components of body composition and baseline characteristics Table S4. Baseline characteristics according to sex‐specific median of the MF‐ratio after propensity score matching Table S5. Risk of CKD development according to body composition indices after propensity score matching Table S6.Sensitivity analysis: Risk of CKD development according to body composition indices using different creatinine conversion formula Figure S1.Study subjects Figure S2.Mutual relationships among components of body composition Figure S3. Restricted cubic spline plot for incident CKD according to MF‐ratio Figure S4. Cumulative Hazards for the incident CKD development according to sex‐specific median of the MF‐ratio in all study subjects (A), normal BMI group (B), overweight group (C), and obese group (D) Figure S5. Subgroup analyses of risk for incident CKD according to high vs. low MF‐ratio groups Figure S6.Frequency of cumulative study visit months [file JCSM-11-726-s001.docx]

**SUPPLEMENTAL MATERIAL**

**Supplemental Table S1.** Risk of CKD development according to baseline characteristics

|  | **Univariable** | |
| --- | --- | --- |
|  | **HR (95% CI)** | ***P*** |
| **MF-ratio (per 1 increase)** | 0.75 (0.69-0.82) | <0.001 |
| **Age (per 1 year increase)** | 1.12 (1.11-1.14) | <0.001 |
| **Sex (male vs. female)** | 1.43 (1.21-1.67) | <0.001 |
| **SBP (per 1 mmHg increase)** | 1.02 (1.01-1.03) | <0.001 |
| **Smoking status (yes vs. no)** | 0.74 (0.63-0.87) | <0.001 |
| **Alcohol intake (yes vs. no)** | 0.64 (0.55-0.75) | <0.001 |
| **Education levels (per increase in level)** | 0.57 (0.50-0.65) | <0.001 |
| **Income levels (per increase in level)** | 0.66 (0.60-0.73) | <0.001 |
| **Hypertension (yes vs. no)** | 2.99 (2.52-3.55) | <0.001 |
| **Diabetes (yes vs. no)** | 2.96 (2.37-3.68) | <0.001 |
| **Physical activity status (yes vs. no)** | 0.87 (0.78-0.97) | 0.013 |
| **eGFR (per 1 mL/min·1.73 m^2^ increase)** | 0.94 (0.93-0.95) | <0.001 |
| **Proteinuria (yes vs. no)** | 1.58 (1.22-2.04) | <0.001 |
| **Total cholesterol (per 1 mg/dL increase)** | 1.01 (1.00-1.01) | <0.001 |
| **CRP (per 1 mg/dL increase)** | 1.20 (1.06-1.35) | 0.004 |
| ***Abbreviations:*** CKD, chronic kidney disease; HR, hazard ratio; CI, confidence interval; MF-ratio, muscle to fat ratio; SBP, systolic blood pressure; eGFR, estimated glomerular filtration rate; CRP, C-reactive protein. | | |

**Supplemental Table S2.** Baseline characteristics (Male vs. Female)

| **Characteristics** | **Total**  **(n = 7,682)** | **Male**  **(n = 3,686)** | **Female**  **(n = 3,996)** | ***P*** |
| --- | --- | --- | --- | --- |
| Body composition |  |  |  |  |
| MF-ratio | 2.9 ± 1.2 | 3.7 ± 1.2 | 2.1 ± 0.6 | <0.001 |
| Muscle mass index (kg/m^2^) | 16.8 ± 1.7 | 17.9 ± 1.5 | 15.8 ± 1.3 | <0.001 |
| Fat mass index (kg/m^2^) | 6.8 ± 2.4 | 5.4 ± 1.7 | 8.0 ± 2.3 | <0.001 |
| BMI, kg/m^2^ | 24.6 ± 3.1 | 24.3 ± 2.9 | 24.9 ± 2.3 | <0.001 |
| WHR | 0.87 ± 0.08 | 0.89 ± 0.06 | 0.86 ± 0.09 | <0.001 |
| Demographic data |  |  |  |  |
| Age, years | 51.4 ± 8.7 | 50.8 ± 8.6 | 51.8 ± 8.8 | <0.001 |
| Smoking status, n (%) | 3,129 (41.2) | 2,935 (80.0) | 194 (4.9) | <0.001 |
| Alcohol status, n (%) | 4,143 (54.4) | 2,991 (81.6) | 1,152 (29.1) | <0.001 |
| Physical activity status, n (%) | 3,796 (50.7) | 1,862 (51.6) | 1,934 (49.8) | 0.058 |
| Physical activity (Mets) | 9095.1 ± 6037.2 | 9374.4 ± 6165.5 | 8837.5 ± 5905.4 | <0.001 |
| SBP, mmHg | 120.9 ± 18.3 | 122.1 ± 16.9 | 119.9 ± 19.5 | <0.001 |
| Education, n (%) |  |  |  | <0.001 |
| Low | 2,282 (29.9) | 643 (17.5) | 1,639 (41.4) |  |
| Intermediate | 4,214 (55.3) | 2,174 (59.3) | 2,040 (51.5) |  |
| High | 1,130 (14.8) | 851 (23.2) | 279 (7.0) |  |
| Income, n (%) |  |  |  | <0.001 |
| Low | 2,357 (31.2) | 880 (24.1) | 1,477 (37.8) |  |
| Intermediate | 2,185 (28.9) | 1,074 (29.4) | 1,111 (28.4) |  |
| High | 3,019 (39.9) | 1,697 (46.5) | 1,322 (33.8) |  |
| Comorbidities, n (%) |  |  |  |  |
| Hypertension | 1,071 (13.9) | 463 (12.6) | 608 (15.2) | <0.001 |
| Diabetes | 493 (6.4) | 261 (7.1) | 232 (5.8) | 0.013 |
| Dyslipidemia | 203 (2.6) | 122 (3.3) | 81 (2.0) | <0.001 |
| CVDs | 105 (1.4) | 53 (1.4) | 52 (1.3) | 0.338 |
| Laboratory data |  |  |  |  |
| eGFR, mL/min/1.73 m^2^ | 93.9 ± 14.2 | 91.4 ± 13.6 | 96.2 ± 14.4 | <0.001 |
| Proteinuria (%) | 581 (7.6) | 299 (8.1) | 282 (7.1) | 0.044 |
| Hemoglobin, g/dL | 13.6 ± 1.6 | 14.8 ± 1.1 | 12.5 ± 1.1 | <0.001 |
| Albumin, g/dL | 4.5 ± 0.3 | 4.6 ± 0.3 | 4.4 ± 0.2 | <0.001 |
| Total cholesterol, mg/dL | 199.1 ± 36.7 | 199.3 ± 36.6 | 198.9 ± 36.9 | 0.676 |
| LDL-C, mg/dL | 119.2 ± 34.5 | 117.9 ± 36.0 | 120.3 ± 32.9 | 0.002 |
| HDL-C, mg/dL | 49.5 ± 11.8 | 47.5 ± 11.3 | 51.4 ± 11.9 | <0.001 |
| Triglyceride, mg/dL | 151.8 ± 108.3 | 169.0 ± 122.3 | 135.9 ± 90.6 | <0.001 |
| Fasting glucose, mg/dL | 92.6 ± 23.2 | 95.1 ± 25.5 | 90.2 ± 20.4 | <0.001 |
| HbA1c, % | 5.8 ± 0.9 | 5.8 ± 0.9 | 5.8 ± 0.8 | 0.039 |
| HOMA-IR | 1.7 ± 1.2 | 1.7 ± 1.2 | 1.8 ± 1.3 | 0.001 |
| CRP [IQR], mg/dL | 0.14 [0.06-0.25] | 0.14 [0.07-0.25] | 0.13 [0.06-0.24] | <0.001 |
| ***Note:*** Data are presented as mean (SD), median [IQR], or number (%).  ***Abbreviations:*** MF-ratio, muscle to fat ratio; BMI, body mass index; WHR, waist to hip ratio; SBP, systolic blood pressure; CVD, cardiovascular disease; eGFR, estimated glomerular filtration rate; LDL-C, low density lipoprotein-cholesterol; HDL-C, high density lipoprotein-cholesterol; HOMA-IR, Homeostatic Model Assessment for Insulin Resistance; CRP, C-reactive protein; SD, standard deviation; IQR, interquartile range. | | | | |

**Supplemental Table S3.** Univariable correlations between components of body composition and baseline characteristics^*^

|  | BMI | | MF-ratio | |
| --- | --- | --- | --- | --- |
|  | *β* | *P* | *β* | *P* |
| Age (year) | -0.02 | 0.080 | -0.10 | <0.001 |
| SBP (mmHg) | -0.19 | <0.001 | -0.10 | <0.001 |
| Total cholesterol (mg/dL) | -0.18 | <0.001 | -0.17 | <0.001 |
| LDL-C (mg/dL) | -0.14 | <0.001 | -0.15 | <0.001 |
| Triglyceride (mg/dL) | -0.21 | <0.001 | -0.08 | <0.001 |
| Albumin (g/dL) | -0.003 | 0.806 | -0.12 | <0.001 |
| HOMA-IR | -0.60 | <0.001 | -0.17 | <0.001 |
| CRP (mg/dL) | -0.05 | <0.001 | -0.03 | 0.021 |
| *Note:* ^*^ Univariable linear regression analyses were performed for body composition indices and clinical variables. *β* represents regression coefficients.  *Abbreviations:* BMI, body mass index; MF-ratio, muscle to fat ratio; SBP, systolic blood pressure; LDL-C, low density lipoprotein-cholesterol; HOMA-IR, Homeostatic Model Assessment for Insulin Resistance; CRP, C-reactive protein. | | | | |

**Supplemental Table S4.** Baseline characteristics according to sex-specific median of MF-ratio after propensity score matching^*^

|  | **Sex-specific MF-ratio** | | | |
| --- | --- | --- | --- | --- |
| **Characteristics** | **Total**  **(n = 3,280)** | **Low**  **(n = 1,640)** | **High**  **(n = 1,640)** | ***P*** |
| Body composition |  |  |  |  |
| MF-ratio | 2.8 ± 0.9 | 2.4 ± 0.6 | 3.2 ± 1.0 | <0.001 |
| Muscle mass index (kg/m^2^) | 16.8 ± 1.6 | 16.4 ± 1.4 | 17.3 ± 1.6 | <0.001 |
| Fat mass index (kg/m^2^) | 6.5 ± 1.6 | 7.2 ± 1.5 | 5.9 ± 1.5 | <0.001 |
| BMI, kg/m^2^ | 24.4 ± 1.8 | 24.6 ± 1.6 | 24.2 ± 1.8 | <0.001 |
| Demographic data |  |  |  |  |
| Age, years | 51.0 ± 8.5 | 51.2 ± 8.6 | 50.9 ± 8.4 | 0.267 |
| Male, n (%) | 1,639 (50.0) | 829 (50.5) | 810 (49.4) | 0.265 |
| Smoking status, n (%) | 1,369 (41.7) | 673 (41.0) | 696 (42.4) | 0.218 |
| Alcohol status, n (%) | 1,794 (54.7) | 889 (54.2) | 905 (55.2) | 0.299 |
| Physical activity status, n (%) | 1,706 (52.0) | 846 (51.6) | 860 (52.4) | 0.325 |
| SBP, mmHg | 120.5 ± 17.3 | 121.0 ± 17.4 | 120.0 ± 17.3 | 0.084 |
| Education, n (%) |  |  |  | 0.648 |
| Low | 935 (28.5) | 475 (29.0) | 460 (28.0) |  |
| Intermediate | 1,835 (55.9) | 911 (55.5) | 924 (56.3) |  |
| High | 510 (15.5) | 254 (15.5) | 256 (15.6) |  |
| Income, n (%) |  |  |  | 0.544 |
| Low | 957 (29.2) | 478 (29.1) | 479 (29.2) |  |
| Intermediate | 929 (28.3) | 449 (27.4) | 480 (29.3) |  |
| High | 1,353 (41.3) | 692 (42.2) | 661 (40.3) |  |
| Comorbidities, n (%) |  |  |  |  |
| Hypertension | 408 (12.4) | 220 (13.4) | 188 (11.5) | 0.101 |
| Diabetes | 205 (6.3) | 100 (6.1) | 105 (6.4) | 0.387 |
| Dyslipidemia | 109 (3.3) | 56 (3.4) | 53 (3.2) | 0.423 |
| CVDs | 38 (1.2) | 22 (1.3) | 16 (1.0) | 0.207 |
| Laboratory data |  |  |  |  |
| eGFR, mL/min/1.73 m^2^ | 93.6 ± 14.3 | 93.7 ± 14.2 | 93.5 ± 14.5 | 0.781 |
| Proteinuria (%) | 225 (6.9) | 108 (6.6) | 117 (7.1) | 0.290 |
| Hemoglobin, g/dL | 13.6 ± 1.6 | 13.7 ± 1.6 | 13.6 ± 1.6 | 0.220 |
| Albumin, g/dL | 4.5 ± 0.3 | 4.5 ± 0.3 | 4.5 ± 0.3 | 0.774 |
| Total cholesterol, mg/dL | 199.7 ± 35.6 | 200.3 ± 35.3 | 199.1 ± 35.8 | 0.340 |
| LDL-C, mg/dL | 120.4 ± 33.6 | 120.9 ± 33.1 | 119.8 ± 34.1 | 0.335 |
| HDL-C, mg/dL | 48.9 ± 11.4 | 48.7 ± 11.2 | 49.1 ± 11.7 | 0.257 |
| Triglyceride, mg/dL | 152.1 ± 103.7 | 153.4 ± 95.7 | 150.8 ± 111.1 | 0.482 |
| Fasting glucose, mg/dL | 92.3 ± 22.8 | 92.4 ± 21.4 | 92.2 ± 24.1 | 0.801 |
| HbA1c, % | 5.7 ± 0.9 | 5.7 ± 0.9 | 5.7 ± 0.9 | 0.907 |
| HOMA-IR | 1.7 ± 1.2 | 1.7 ± 1.2 | 1.7 ± 1.2 | 0.702 |
| CRP [IQR], mg/dL | 0.14 [0.07-0.24] | 0.14 [0.07-0.25] | 0.13 [0.06-0.23] | 0.846 |
| ***Note:*** Data are presented as mean (SD), median [IQR], or number (%).  ^*^Covariates for propensity score matching was age, sex, SBP, BMI, education level, history of hypertension, diabetes, cardiovascular events, or dyslipidemia, physical activity, hemoglobin, albumin, total cholesterol, HbA1c, CRP, eGFR, and proteinuria. Subjects were classified into two groups by MF-ratio: high vs. low. The propensity score was determined using binary logistic regression with greedy nearest neighbor matching technique without replacement. A caliper of 0.2 times the standard deviation was used. Subjects with high MF-ratio were matched to those with low MF-ratio.  ***Abbreviations:*** MF-ratio, muscle to fat ratio; BMI, body mass index; WHR, waist to hip ratio; SBP, systolic blood pressure; CVD, cardiovascular disease; eGFR, estimated glomerular filtration rate; LDL-C, low density lipoprotein-cholesterol; HDL-C, high density lipoprotein-cholesterol; HOMA-IR, Homeostatic Model Assessment for Insulin Resistance; CRP, C-reactive protein; SD, standard deviation; IQR, interquartile range. | | | | |

**Supplemental Table S5** Risk of CKD development according to body composition indices after propensity score matching

|  | **Before PSM** | | **After PSM** | |
| --- | --- | --- | --- | --- |
|  | **HR (95% CI)*** | ***P*** | **HR (95% CI)†** | ***P*** |
| **BMI** |  |  |  |  |
| per 1 kg/m^2^ increase | 1.04 (0.99-1.08) | 0.059 | 1.01 (0.93-1.08) | 0.833 |
| high vs. low | 1.10 (0.93-1.31) | 0.245 | 0.82 (0.64-1.05) | 0.113 |
| **MF-ratio** |  |  |  |  |
| per 1 increase | 0.86 (0.77-0.96) | 0.008 | 0.85 (0.74-0.98) | 0.026 |
| high vs. low | 0.83 (0.70-0.98) | 0.031 | 0.84 (0.71-0.98) | 0.037 |
| ***Note:***  ***** Adjusted for age, sex, SBP, smoking status, alcohol intake, education levels, income levels, history of hypertension or diabetes, and physical activity,  eGFR, proteinuria, total cholesterol, and CRP.  **†** Covariates for propensity score matching was age, sex, SBP, BMI, education level, history of hypertension, diabetes, cardiovascular events, or dyslipidemia,  physical activity, hemoglobin, albumin, total cholesterol, HbA1c, CRP, eGFR, and proteinuria. Subjects were classified into two groups by MF-ratio: high vs. low.  The propensity score was determined using binary logistic regression with greedy nearest neighbor matching technique without replacement. A caliper of 0.2 times  the standard deviation was used. Subjects with high MF-ratio were matched to those with low MF-ratio.  ***Abbreviations:*** CKD, chronic kidney disease; HR, hazard ratio; CI, confidence interval; BMI, body mass index; MF-ratio, muscle to fat ratio; SBP, systolic blood  pressure; eGFR, estimated glomerular filtration rate; CRP, C-reactive protein. | | | | |

**Supplemental Table S6** Sensitivity analysis: Risk of CKD development according to body composition indices using different creatinine conversion formula*****

|  | **Model 1** | | **Model 2** | | **Model 3** | | **Model 4** | |
| --- | --- | --- | --- | --- | --- | --- | --- | --- |
|  | **HR (95% CI)** | ***P*** | **HR (95% CI)** | ***P*** | **HR (95% CI)** | ***P*** | **HR (95% CI)** | ***P*** |
| **BMI** |  |  |  |  |  |  |  |  |
| per 1 kg/m^2^ increase | 1.05 (1.02-1.08) | 0.002 | 1.07 (1.04-1.10) | <0.001 | 1.05 (1.02-1.09) | 0.001 | 1.03 (0.98-1.06) | 0.066 |
| high vs. low | 1.16 (0.97-1.39) | 0.101 | 1.26 (1.05-1.51) | 0.012 | 1.11 (0.91-1.35) | 0.294 | 1.01 (0.83-1.22) | 0.945 |
| **MF-ratio** |  |  |  |  |  |  |  |  |
| per 1 increase | 0.81 (0.74-0.88) | <0.001 | 0.80 (0.71-0.89) | <0.001 | 0.84 (0.75-0.95) | 0.005 | 0.86 (0.76-0.97) | 0.016 |
| high vs. low | 0.68 (0.57-0.82) | <0.001 | 0.78 (0.65-0.94) | 0.008 | 0.81 (0.66-0.99) | 0.043 | 0.81 (0.66-0.99) | 0.039 |
| ***Note:***  ***** Creatinine levels were adjusted using a conversion equation previously reported^1,2^  Model 1: Unadjusted model  Model 2: Adjusted for age and sex  Model 3: Adjusted for Model 2 + SBP, smoking status, alcohol intake, education levels, income levels, history of hypertension or diabetes, and physical activity  Model 4: Adjusted for Model 3 + eGFR, proteinuria, total cholesterol, and CRP  ***Abbreviations:*** CKD, chronic kidney disease; HR, hazard ratio; CI, confidence interval; BMI, body mass index; MF-ratio, muscle to fat ratio; SBP, systolic blood  pressure; eGFR, estimated glomerular filtration rate; CRP, C-reactive protein.  ***Reference:***  1. ClinCalc.com. IDMS to conventional serum creatinine [Internet]. ClinCalc LLC [cited 2008 Jun 12]: Available from: https://clincalc.Com/kinetics/idms.Aspx.  2. Lee C, Yun HR, Joo YS, Lee S, Kim J, Nam KH, et al. Framingham risk score and risk of incident chronic kidney disease: A community-based prospective cohort study. Kidney Res Clin Pract 2019;38:49-59. | | | | | | | | |


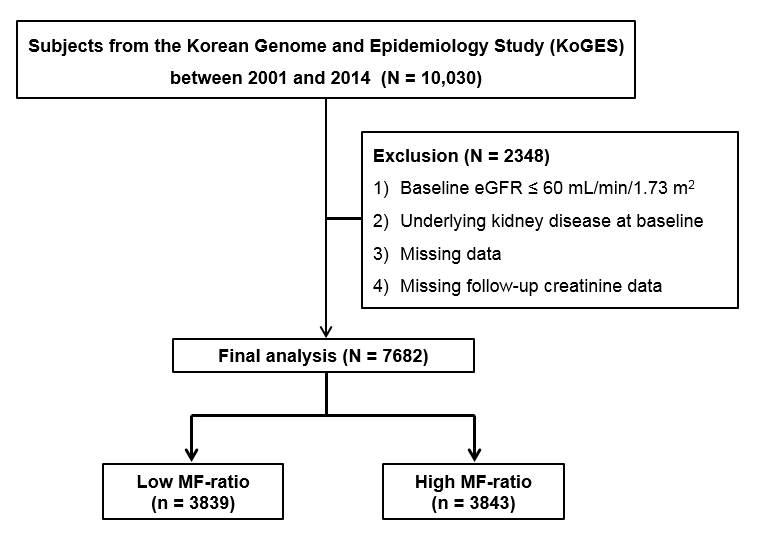


**Supplemental Figure S1.** Study subjects

***Abbreviations:*** eGFR, estimated glomerular filtration rate; MF-ratio, muscle to fat ratio.


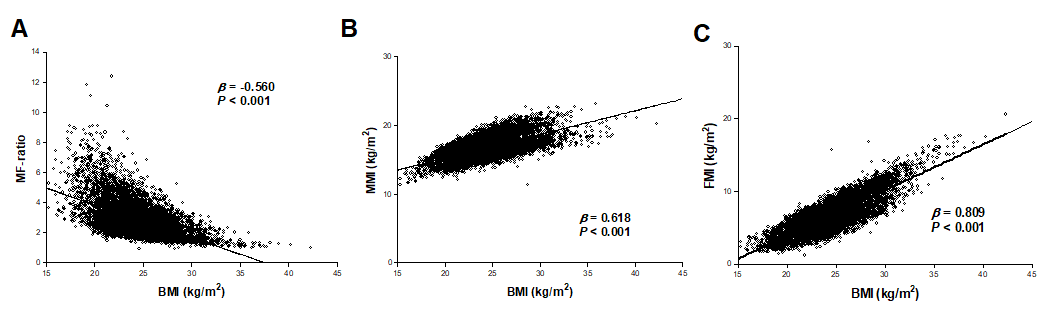


**Supplemental Figure S2.** Mutual relationships among components of body composition

***Abbreviations:*** BMI, body mass index; MF-ratio, muscle to fat ratio; MMI, muscle mass index; FMI, fat mass index


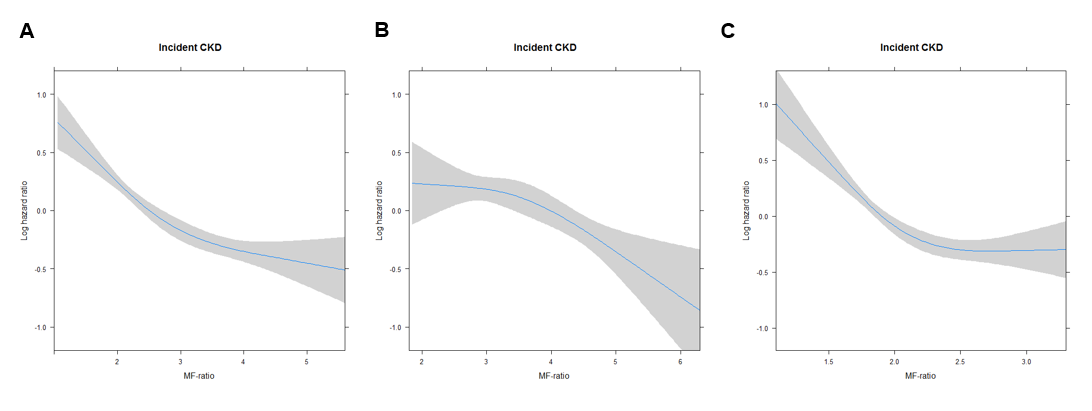


**Supplemental Figure S3.** Restricted cubic spline plot for incident CKD according to MF-ratio (A) in both female and male, (B) male, and (C) female participants

***Note:*** Blue line represents HR and gray surface area represents 95% CI.

***Abbreviations:*** MF-ratio, muscle to fat ratio; HR, hazard ratio; CKD, chronic kidney disease; CI, confidence interval.


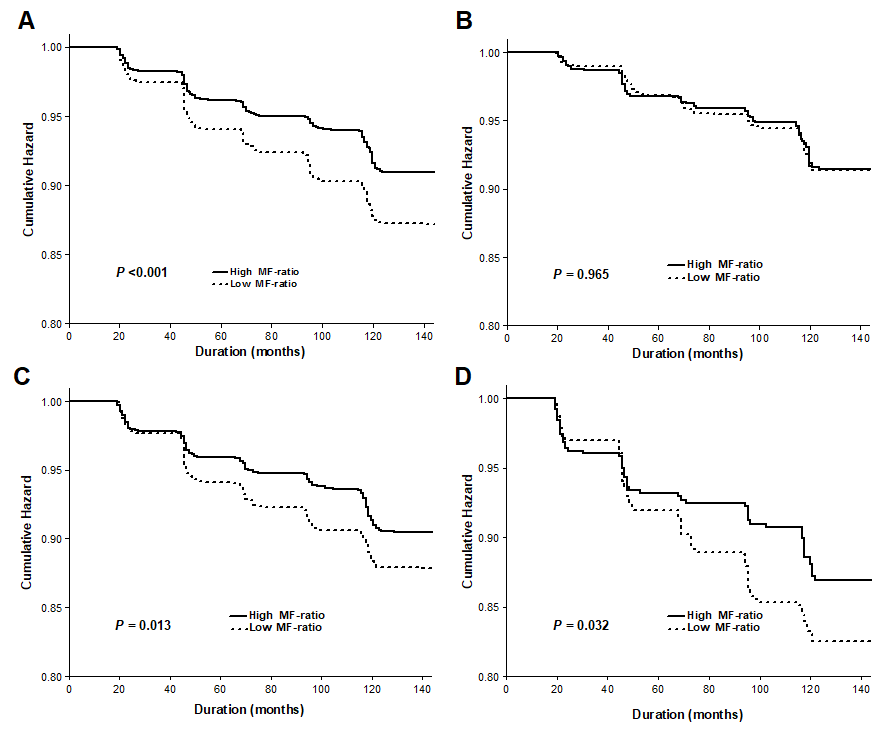


**Supplemental Figure S4.** Cumulative Hazards for the incident CKD development according to sex-specific median of MF-ratio in all study subjects (A), normal BMI group (B), overweight group (C), and obese group (D)

***Note:*** WHO obesity classification for Asian population was used; normal (BMI <23.0 kg/m^2^), overweight (BMI 23.0-27.4 kg/m^2^), and obese (BMI ≥27.5 kg/m^2^).

***Abbreviations:*** CKD, chronic kidney disease; MF-ratio, muscle to fat ratio; BMI, body mass index.


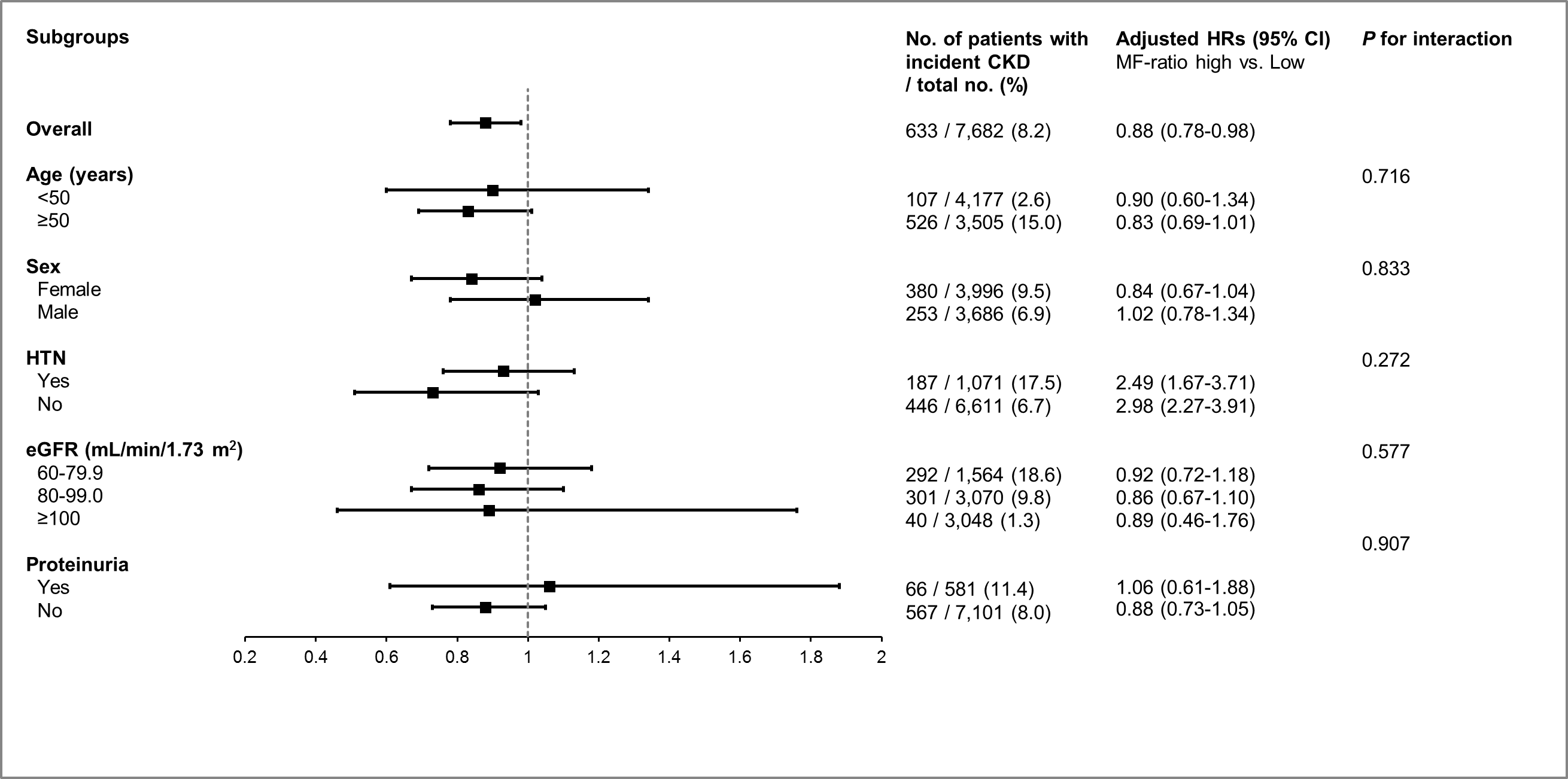


**Supplemental Figure S5.** Subgroup analyses of risk for incident CKD according to high vs. low MF-ratio groups

***Abbreviations:*** HR, hazard ratio; CI, confidence interval; CKD, chronic kidney disease; MF-ratio, muscle to fat ratio; HTN, hypertension; eGFR, estimated glomerular filtration rate.


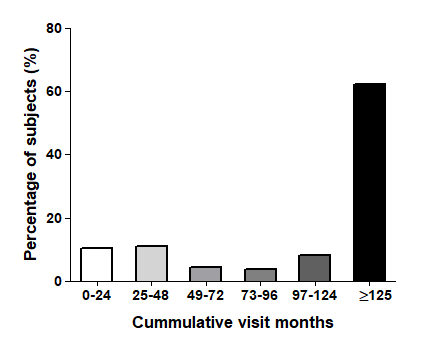


**Supplemental Figure S6.** Frequency of cumulative study visit months
